# EncephalApp Stroop Test for covert hepatic encephalopathy screening in Tunisian cirrhotic patients

**DOI:** 10.12688/f1000research.121781.1

**Published:** 2022-06-21

**Authors:** Lamine Hamzaoui, Moufida Mahmoudi, Ghanem Mohamed, Hanene Elloumi, Asma Laabidi, Jalel Boubaker, Mona Boudabbous, Nabil Tahri, Imen Jemni, Leila Safer, Taieb Jomni, Hedi Douggui, Dorra Trad, Dalila Gargouri, Shema Ayadi, Radhouane Debbeche, Sami Belhouchet, Ridha Marouani, Imed Cheikh, Mohamed Nabil Abdelli

**Affiliations:** 1 Gastroenterology, Mohamed Taher Maamouri Hospital, Tunis El Manar University, Faculty of Medicine of Tunis, Nabeul, Tunisia; 2 Gastroenterology, Military Hospital of Tunis, Tunis El Manar University, Faculty of Medicine of Tunis, Tunis, Tunisia; 3 Gastroenterology, Habib Bougatfa Hospital, Tunis El Manar University, Faculty of Medicine of Tunis, Bizerte, Tunisia; 4 Gastroenterology, La Rabta Hospital A, Tunis El Manar University, Faculty of Medicine of Tunis, Tunis, Tunisia; 5 Gastroenterology, Hedi Chaker Hospital, Faculty of Medicine of Sfax, Sfax, Tunisia; 6 Gastroenterology, Fattouma Bourguiba University Hospital, Monastir, Monastir, Tunisia; 7 Gastroenterology, La Marsa Internal Security Forces Hospital, Tunis El Manar University, Faculty of Medicine of Tunis, Tunis, Tunisia; 8 Gastroenterology, Habib Thameur Hospital, Tunis El Manar University, Faculty of Medicine of Tunis, Tunis, Tunisia; 9 Gastroenterology, Hospital Charles Nicolle, Tunis, Tunis El Manar University, Faculty of Medicine of Tunis, Tunis, Tunisia; 10 Gastroenterology, Jendouba Hospital, Jendouba, Tunisia; 11 Gastroenterology, Kasserine Hospital, Kasserine, Tunisia

**Keywords:** Cirrhosis, Tool, Subclinical hepatic encephalopathy, West Haven Criteria, Medical apps

## Abstract

**Background: **Covert hepatic encephalopathy (CHE) is underdiagnosed and is difficult to detect. The EncephalApp Stroop test is validated for its screening. The aim of the study was to define Tunisian norms for the test based on healthy controls norms and to estimate the prevalence of CHE in cirrhotic Tunisian patients.

**Methods:** A prospective, multicenter, cross‐sectional study was conducted. Ambulatory or hospitalized cirrhotic patients aged 40 years and over were recruited at 11 centers. Healthy subjects aged 40 years and over were recruited at 8 centers. We used a translated Arabic version of the streamlined EncephalApp Stroop test. The task has two components: “Off” and “On” state depending on the discordance or concordance of the stimuli.

**Results:** 142 patients were included. The mean age was 57.26 years [40–86]. 40 (28.17%) of cirrhotic patients who were included were diagnosed as having a minimal hepatic encephalopathy or CHE. Among the ineligible patients, 22 had overt hepatic encephalopathy. If we consider these patients, the overall prevalence rate of CHE was around 24.39% in cirrhotic patients. It was more frequent in women (34.21% *vs* 25.96%), and in patients whose level of school education is between 6 and 13 years. Its prevalence does not appear to be affected by gender, MELD score, etiology of cirrhosis and age group of patients, as these variables were independent with respective p according to the chi-square test 0.413; 0.736; 0.663 and 0.1. The stroop times (On / Off and On + Off) correlated significantly with each other, are associated significantly and positively with age (respective Pearson coefficients: 0.578; 0.567 and 0.6). The more the age increases, the more the stroop response times increases (p > 10 ^-3^).

**Conclusions:** EncephalApp Stroop test was an efficient screening tool for CHE in Tunisian cirrhotic patients.

## Abbreviations

ANT: Animal Naming test

CFF: Critical Flicker Frequency

CHE: covert hepatic encephalopathy

CRT: Continuous Reaction Time (CRT)

HE: hepatic encephalopathy

ICT: inhibitory control test

ISHEN: International Society for Hepatic Encephalopathy and Nitrogen Metabolism

MELD: Model for End-Stage Liver Disease

NCT-B: Number connection test- B (NCT-B)

OHE: overt hepatic encephalopathy

PHES: Psychometric Hepatic Encephalopathy Score

SDT: serial dotting test

SPT: standard psychometric test

StE: Stroop Encephal-App

## Introduction

Covert hepatic encephalopathy (CHE) is a part of the spectrum of neurocognitive impairment in cirrhosis. ^1^^,^^2^ Patients with CHE have slow alterations of their psychomotor and neuropsychiatric functions, ^3^ without any clinical evidence of cognitive changes, making its diagnosis a veritable challenge. ^4^^–^^7^ Despite representing the earliest and mildest form of hepatic encephalopathy (HE), ^3^ CHE is associated with increased risks of progression to overt hepatic encephalopathy (OHE), hospitalization and mortality. ^3^^,^^5^^,^^8^^,^^9^ It is also associated with impaired employment, driving capability and quality of life. ^8^^–^^14^ CHE treatment can improve quality of life and prevent progression to OHE. ^15^^–^^18^ However, this condition represents the most underdiagnosed form of HE and its treatment is not part of the standard of care. ^19^^,^^20^ Currently, there is several validated testing modalities to detect this complication. Stroop Encephal-App (StE) test, a sensitive test for the detection of cognitive impairment in CHE, ^6^^,^^21^^,^^22^ is an attractive option for point of care testing strategy of CHE in cirrhotic patients. It evaluates cognitive flexibility and psychomotor speed. ^21^^,^^22^ It is a simple, short, valid and reliable test. ^6^^,^^21^^,^^22^ The aims of the current study were to define Tunisian norms for the StE test based on healthy controls norms and to estimate the prevalence of CHE in cirrhotic Tunisian patients.

## Methods

### Study design and oversight

This was a prospective, multicenter, cross-sectional study conducted from May 14, 2018, to October 2, 2020, at 11 centers across Tunisia. The study was designed and led by a steering committee that included academic investigators and representatives from AlphaSigma. A contract research organization (Poseidoncro) performed database development, centers monitoring and data analysis. The study was conducted in accordance with the International Council for Harmonization guidelines for Good Clinical Practice and the 1975 Declaration of Helsinki as well as local regulations and was approved by institutional review boards and ethics committees at each trial center. Written informed consent was obtained from all participants.

### Study population

Ambulatory or hospitalized cirrhotic patients aged 40 years and over were recruited at 11 centers. We excluded patients with history or evidence at screening of OHE . Other exclusion criteria were illiterate patient, inability to perform the test due to mental or physical disabilities, red-green color blindness, psychoactive medications use and history of transjugular intrahepatic portosystemic shunt or surgical portosystemic shunt.

Healthy subjects aged 40 years and over were recruited at 8 centers among members of the medical and nursing staff, their relatives, and friends. Exclusion criteria were illiterate patients, inability to perform the test due to mental or physical disabilities, red-green color blindness and psychoactive medications use.

### Instrument and procedure

We used a translated Arabic version of the streamlined EncephalApp stroop test. The test was carried out on an android tablet with a 10-inch screen. Briefly, the task has two components: “Off” and “On” state depending on the discordance or concordance of the stimuli. Both components were administered after two training runs. Five correct runs were required for each component. The specific results of EncephalApp are as follows: 1) total time for five correct runs in the off state (Off time); 2) number of runs needed to complete the five correct Off runs; 3) total time for five correct runs in the on state (On time); and 4) number of runs needed to complete the five correct on runs. The Off Time+On Time was the ultimate measure used to diagnose the presence of CHE.

The subjects who responded to the eligibility criteria were sorted to take the test. The data was collected through a case report form in which we gathered information about sex, age, educational level, the etiology of the cirrhosis, the alcohol or tobacco use, and the Model for End-Stage Liver Disease (MELD) score, as well as the test results for each participant.

### Statistical analysis

A sample of 124 healthy subjects was deemed as representative of the general population with a 80% confidence level and a 95% confidence range. A non-proportionate quota sampling strategy was used to enroll the healthy subjects. Quotas were set regarding age categories, sex, and education level. Age, sex, and education level were selected for quota sampling because they are important predictors of Stroop test performance. To guarantee the presence of the required number of healthy subjects, and to make sure of the equitability of the samples between the different centers, we added 20 healthy subjects to the study. A total of 18 participants were recruited at each of the 8 centers following a quota sampling matrix of 3×2×3 cells.

## Results

### Validation

All the norms defined in the Tunisian population after adjustment for age, gender, and education level are shown in Table 1.

**Table 1.** Tunisian norms of the stroop on + stroop off time variable in healthy subjects.

Study level		<6		6–13		>13	
Gender		Male	Female	Male	Female	Male	Female
40-50 years	Mean	204.66	265.22	182.07	193.21	166.43	177.61
	SE	71.84	149.47	66.86	38.18	28.04	25.71
	Min-Max	152.23-369.38	149.29-582.08	133.14-334.44	142.24-265.26	138.74-210.22	145.93-210.91
50-60 years	Mean	227.5	262.5	210.26	183.07	210.75	207.09
	SE	59.40	88.77	49.66	21.06	61.62	78.69
	Min-Max	169.41-334.65	179.36-403.68	148.74-306.09	162.06-225.42	141.75-331.61	151.05-383.88
> 60 years	Mean	321.15	271.06	243.04	274.58	220.64	196.27
	SE	89.39	101.95	54.3	122.4	47.01	45.05
	Min-Max	213.03-465.22	153.42-464.15	185.57-344.16	134.77-503.49	138.0-262.87	138.1-267.52

### Descriptive statistics of cirrhotic patients

Among 369 recruited cirrhotic patients, 142 were included and 227 were not eligible. Almost half of the cirrhotic patients recruited are illiterate (49.32%) ( Table 2).

**Table 2.** Non-eligibility criteria.

Non eligibility criteria	Number
Illiterate	182
Non consenting	7
OHE	22
Muscular or neurologic pathology	4
Intoxication	1
Low visual acuity	3
Dyschromatopsia	1
Cerebral vascular accident	6

*Regions*

Cirrhotic patients are distributed into 11 centers and 6 regions ( Table 3).

**Table 3.** Recruited patients per region.

Region	Investigation center	Recruited patients (n)	Local prevalence of CHE (%)	Global prevalence of CHE (%)
Tunis	Military Hospital	19	28.79	13.38
	La Rabta A Hospital	18	27.27	12.68
	Hospital of the internal security forces of La Marsa	11	16.67	7.75
	Charles Nicolle Hospital	8	12.12	5.63
	Habib Thameur Hospital	10	15.15	7.04
East North	Bizerte Hospital	6	14.29	4.22
	Nabeul Hospital	36	85.71	25.35
West-north	Jendouba Hospital	5	100	0.89
East-center	Monastir Hospital	13	100	9.16
Sfax	Hedi Chaker Hospital	14	100	9.86
West-center	Kasserine Hospital	2	100	1.41

*Baseline characteristics*

**Figure 1.**
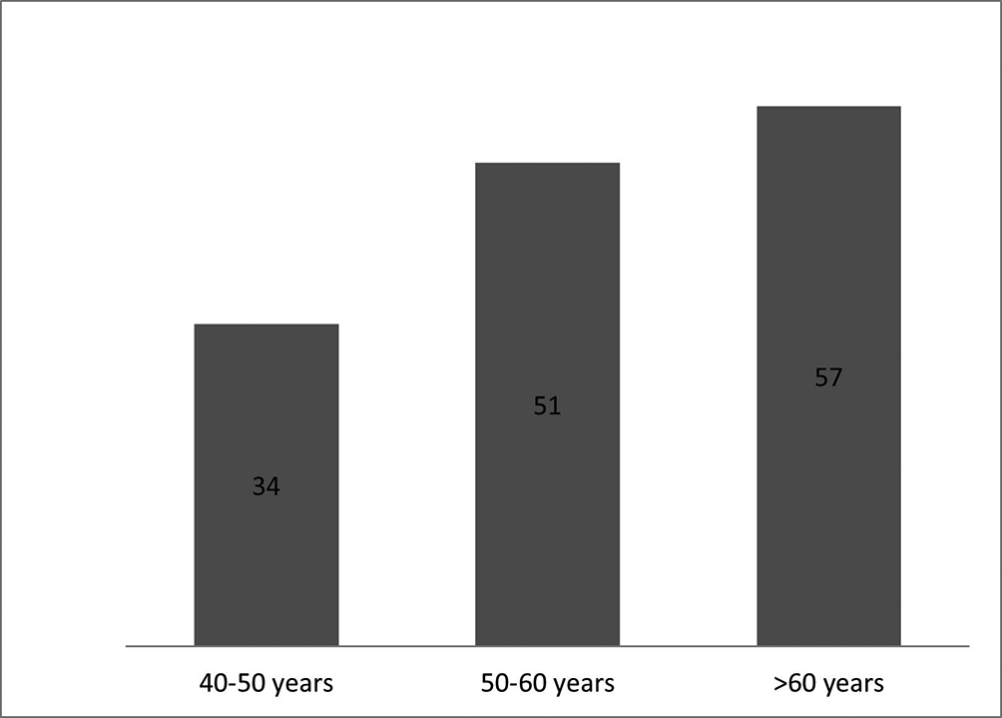
Number of included patients per age groups.

Baseline characteristics are detailed in Table 4. The population of cirrhosis patients meeting the study selection criteria is predominantly male with an M/F sex ratio of 2.74. The mean age was 57.26 years (± DS 9.34 years) [40-86]. The North region has the population with the highest average age of 60.048 years (± 7.24), while the region of Sfax has the youngest population with an average of 53.21 years (± 9.53) ( Table 5). The patients were allocated on three age groups, as studied in the validation phase ( Figure 1).

**Table 4.** Baseline characteristics.

Age (Y)	
Mean	57.26
Sex ratio	2.74
Level of school Education n (%)	
<6	31 (21.83)
6-13	92 (64.79)
>13	19 (13.38)
Habits	
Alcohol consumption n (%)	
No	131 (92.25)
Occasional	6 (4.22)
Heavy	5 (3.52)
Drug use n (%)	5 (3.52)
Etiology of cirrhosis n (%)	
Viral hepatitis B, C	77 (55.8)
Unknown	20 (14.49)
NASH	13 (9.49)
Other	27 (20.22)

**Table 5.** Mean age per region.

Variable	Tunis	East-north	West_north	East-center	Sfax	West-center
Min-Max	40-86	43-74	44-66	40-74	40-68	49-61
Mean	57.01	60.04	57.8	53.93	53.21	55
SE	9.83	7.24	7.38	10.26	9.53	6.00

### Prevalence of CHE

**Figure 2.**
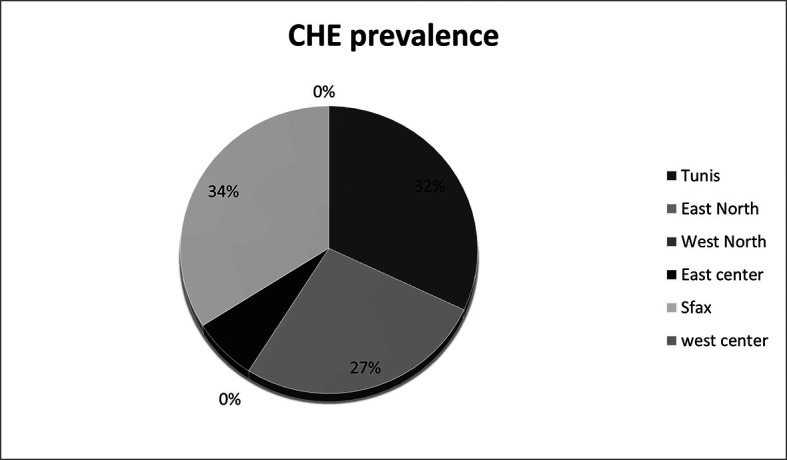
CHE prevalence in asymptomatic patients according to regions.

**Figure 3.**
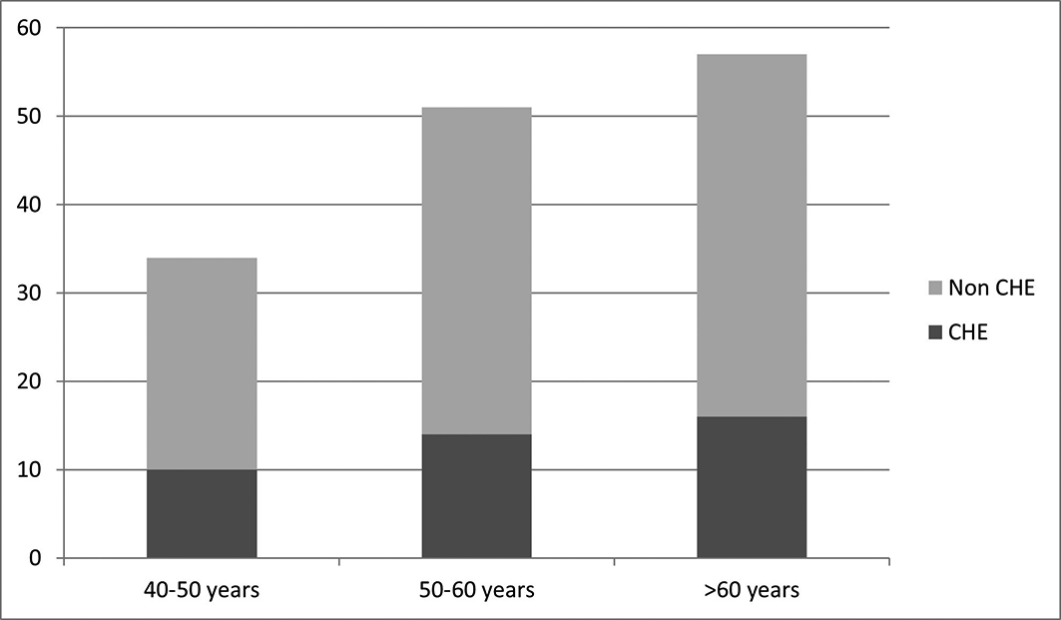
CHE prevalence in age groups.

40 (28.17%) of cirrhotic patients who were included and passed the Stroop test were diagnosed as having MHE/CHE. Among the ineligible patients, 22 had OHE and therefore most likely do not have CHE. If we consider these patients, the overall prevalence rate of CHE was around 24.39% in cirrhotic patients. The highest prevalence rate (29.73%) was recorded in the region of Tunis ( Figure 2). CHE is more frequent in women (34.21% *vs* 25.96%), and in patients whose level of school education is between 6 and 13 years (70% *vs* 20% in patients with education level <6 years and 10% in patients with education level >13 years). CHE prevalence according to age groups is represented in Figure 3.

### Analytic statistics

Prevalence of CHE does not appear to be affected by gender, MELD score, etiology of cirrhosis and age group of patients, as these variables were independent with respective p according to the chi-square test 0.413; 0.736; 0.663 and 0.1.

The stroop times (On/Off and On + Off) correlated significantly with each other, are correlated significantly and positively with age (respective Pearson coefficients: 0.578; 0.567 and 0.6). The more the age increases, the more the stroop response times increases (p > 10 ^-3^). On the other hand, the level of education is negatively and significantly correlated with the stroop responses times (−0.611; −0.491 and −0.584 respectively; p ≤ 10 ^-3^).

## Discussion

CHE is a cognitive impairment in cirrhotic patients. It is characterized by abnormalities in attention and psychomotor speed. ^1^ However, it is difficult to diagnose by using simple clinical examination. ^1^^,^^23^ Its diagnosis is, also hampered by the lack of tests that can be applied at point of care without psychological expertise. ^23^ CHE stills underdiagnosed. About 38% of American physicians never test for HE because of time, costs or requirement of trained staff and equipment. ^20^ Another survey from India showed that only 6.3% of physicians screened their patients for minimal hepatic encephalopathy. ^19^

The latest American and European Associations for the Study of Liver Diseases practice guidelines recommended that CHE should be diagnosed by neuropsychological, neurophysiological, or computerized testing. ^4^ According to the International Society for Hepatic Encephalopathy and Nitrogen Metabolism (ISHEN) consensus, neuropsychological tests, such as Psychometric Hepatic Encephalopathy Score (PHES), Critical Flicker Frequency (CFF), Continuous Reaction Time (CRT), EncephalApp, and Animal Naming test (ANT) have had validation and could be recommended for investigating CHE. The combination of two tests and more to establish the diagnosis of CHE is discouraged. ^24^

Some CHE diagnostic tests like PHES are time consuming and necessitate trained physicians. ^21^^,^^25^ The application of rapid and simple point-of-care tests like ANT or StE could be of great benefit for the screening of CHE. ^6^^,^^24^^,^^26^ StE Assesses cognitive flexibility and psychomotor speed in paper-pencil and electronic formats through a conflict between word reading and color naming. ^22^^,^^27^ Since 2013, Bajaj *et al.*, found that the StE was able to detect cognitive dysfunction with good discriminative validity and test-retest reliability in cirrhosis. ^21^ Later studies had confirmed the face and external validity and test-retest reliability of this test. ^6^^,^^22^

The StE is easy to administer, quick to teach to subjects, and simple to score and interpret. Its use is appropriate for CHE screening. ^21^^,^^27^ Moreover, when we consider the financial impact of untreated CHE, the cost effectiveness ratio is in favor of StE. ^28^^,^^29^ This test is available on iTunes for free download in different languages. ^21^ The major limitation of its use is the requirement a compatible device to be applicable.

The stroop test consists to measure psychomotor function (time required to complete the tasks), cognitive flexibility (On time - off time) and errors committed (number of runs required). ^21^ It was found that psychomotor function was more predictive than errors committed and cognitive flexibility in differentiating patients with CHE and without CHE. ^30^^–^^32^ The consistent differentiators between affected and unaffected patients with CHE are the indices of psychomotor speed and reaction time (Off time + On time) and not measures of cognitive flexibility (On time - Off times) or those of accuracy (number of runs required). ^32^ In fact, it was demonstrated that On Time + Off Time was the best element to discriminate CHE patients among cirrhotic patients. ^21^^,^^22^ The cutoff values retained for Off time + On time in the US cirrhotic patients were 274.9, 195.9 and 190 seconds based on PHEES, SPT or ICT. ^6^^,^^21^^,^^22^ In Chinese patients, the cutoff value retained for Off time + On time was 186.63 seconds based on PHES. ^25^ Additional studies are indicated to validate diagnostic cutoffs. ^24^ The sensitivity and the specificity of StE was ranging from 72% to 88% and 54% to 81% respectively according to the reference test and to the study: PHSPT, standard psychometric test (SPT) or inhibitory control test (ICT). ^6^^,^^21^^,^^22^^,^^25^ The first study conducted by Bajaj *et al*, showed that the EncephalApp had a sensitivity of 78% and specificity of 90% for diagnosing MHE when using the PHES as the reference standard. ^21^ A subsequent study realized by the same team, found 80% sensitivity and 81% specificity for the diagnosis of CHE in patients without prior OHE. ^22^ A US multicenter study reported that the EncephalApp had a sensitivity of 80% and of 70% and a specificity of 61% and 54% based on PHES and ICT respectively. ^6^ A more recent Chinese study demonstrated 86% sensitivity and 59% specificity. ^25^

In our study, because of the lack of Tunisian norms, we first recruited healthy controls for the diagnosis of CHE. The cutoff values were to retain the diagnosis of CHE and were obtained according to the values recorded in healthy subjects (+ two Standard deviation) after adjustment for age, gender, and education level. The lack of a reference standard test may constitute one limitation in our study although it have been demonstrated that the use of PHES and StE, whether alone or in combination, were found to be equivalent for screening CHE, which indicates that single testing with the StE is sufficient for screening CHE. ^33^ Recently, a Chinese study suggested that the combination of StE and two subtests of PHES (Number connection test- B (NCT-B) and serial dotting test (SDT)) is a good diagnostic tool for CHE in cirrhotic patients with 97.7% sensitivity, 79.3 specificity and 86.9% accuracy. This combination is also time saving compared to PHES. ^7^

The prevalence of CHE in cirrhotic patients varies between 20% and 80% around the world. ^5^^,^^19^^,^^34^^–^^38^ Epidemiologic studies regarding CHE are lacking in Tunisia. There is no normalized and validated data for appropriate diagnostic test for MHE. Our study is the first one screening CHE in Tunisian cirrhotic patients using StE test. The prevalence of CHE was 24.39%.

Several factors such as age, severity of liver disease, alcoholic etiology, history of OHE and portosystemic shunts could influence the prevalence of CHE. ^39^ It was observed that age and education levels were correlated with the risk of CHE diagnosis based on StE. Gender influence on StE outcomes was however controversial. ^6^^,^^21^^,^^22^^,^^25^ Patients with alcoholic liver disease were more likely to perform worse StE scores. ^21^^,^^40^ These data indicates that contrary to the benefic effect of education on cognitive functions, age and alcohol could have a negative impact on cognitive ability. ^21^^,^^25^ Smoking was also considered as a risk factor for MHE. ^41^^,^^42^ In our study, CHE was more frequent in women, elderly patients and in patients whose level of school education is between 6 and 13 years compared to patients with education level <6 years and >13 years). None of our patients had alcoholic cirrhosis.

Concerning the severity of the liver disease, it was established that the application is able de discriminate patients from the early to advanced stage of cirrhosis. ^14^^,^^43^^,^^44^ The StE times were correlated with MELD scores ^21^^,^^22^ and were worse in previous OHE patients. ^6^^,^^21^^,^^22^^,^^25^ According to an American multicenter study, poor StE performance based on ICT predicted OHE independently of the MELD (Hazard ratio: 4.1). ^6^ It has been observed that patients with previous OHE did not improve on repeated testing contrary to patients without previous OHE. ^43^^,^^44^ The StE could also be considered for therapeutic trials. ^21^

In our study, the prevalence of CHE does not appear to be affected by gender, MELD score, etiology of cirrhosis and age group of patients (p = 0.413; 0.736; 0.663 and 0.1 respectively). The stroop times (On/Off and On + Off) were significantly and positively correlated with age (respective Pearson coefficients: 0.567 and 0.6). The more the age increases, the more the stroop response times increases (p > 10 ^-3^). On the other hand, the level of education is negatively and significantly correlated with the stroop responses times (p ≤ 10 ^-3^).

Our study is the first one estimating the prevalence of CHE in Tunisia. There are some limitations to the present study. Firstly, there is the lack cutoff values of StE based on a reference standard test and secondly, a relatively small sample size of patients was recruited in this study. Further multicenter studies are required to determine the cutoff values of StE for the diagnosis of CHE and the prevalence of CHE in Tunisian patients.

## Conclusion

EncephalApp Stroop test, validated in several studies, was an efficient screening tool for CHE in Tunisian cirrhotic patients.

## Data availability

All data underlying the results are available as part of the article and no additional source data are required.
