# Supplementary material for: EncephalApp Stroop Test for covert hepatic encephalopathy screening in Tunisian cirrhotic patients
Source: F1000Res. 2023 Sep 1;11:686. Originally published 2022 Jun 21. [Version 2] doi: 10.12688/f1000research.121781.2 (PMC10521086; doi:10.12688/f1000research.121781.2)
Supplement: Peer review history

# Reviewer report: Reviewer response for version 2

**DOI:** 10.5256/f1000research.155224.r203047

**Published:** 2023-09-12

**Authors:** Helene Larrue

**Affiliations:** 1 Service d'Hépatologie, CHU de Toulouse, Université Toulouse III - Paul Sabatier, Toulouse, France

Thank you to the authors for answering the questions. The article is now improved.

Is the work clearly and accurately presented and does it cite the current literature?

Yes

If applicable, is the statistical analysis and its interpretation appropriate?

Partly

Are all the source data underlying the results available to ensure full reproducibility?

Partly

Is the study design appropriate and is the work technically sound?

No

Are the conclusions drawn adequately supported by the results?

Yes

Are sufficient details of methods and analysis provided to allow replication by others?

No

Reviewer Expertise:

chronic liver disease, portal hypertension

I confirm that I have read this submission and believe that I have an appropriate level of expertise to confirm that it is of an acceptable scientific standard.

# Reviewer report: Reviewer response for version 1

**DOI:** 10.5256/f1000research.133680.r146617

**Published:** 2022-08-22

**Authors:** Helene Larrue

**Affiliations:** 1 Service d'Hépatologie, CHU de Toulouse, Université Toulouse III - Paul Sabatier, Toulouse, France

In the present study, the authors aimed both to define specific norms for Stroop Encephal-App test (StE) and to estimate the prevalence of covert hepatic encephalopathy (CHE) in cirrhotic Tunisian population. This was a prospective multicenter, cross-sectional study with healthy controls to help define norms.

 A total of 142 cirrhotic patients and 124 healthy subjects were recruited. Tunisian norms of the stroop on + off time variable was defined in healthy subjects, in subgroups by age, gender and study level.

 The prevalence of CHE in cirrhotic patients was 28.17% and was more frequent in women (34.21 vs 25.96%) and in patients whose level of school education was between 6 – 13 years (70% vs 20% in patients with education level <6 years).

 This work validated the use of EncephalApp Stroop test as an efficient tool to diagnose CME. Also this is the first study to estimate the prevalence of CHE in cirrhotic Tunisian population.

 The major concern is the lack of definition of cutoff values of StE to define CHE as the authors didn’t use any reference test. So, we don’t know whether the cutoff values (based on healthy patients) used to classify patients as having CHE or not were right. Thus, neither of the study's objectives were properly met.

 In addition, the sample size is small and near 61% of patients recruited weren’t included mainly because of illiteracy.

 In the section “Prevalence of CHE”, it’s said CHE is more frequent in women… but there are no statistical tests proving it (no p-value).

 In table 3, it’s not very clear the difference between local prevalence of CHE and global prevalence of CHE, please clarify this.

 In the section “Analytic statistics”, it’s written “The stroop times correlated significantly with each other are correlated significantly and positively with age”, please rephrase.

Is the work clearly and accurately presented and does it cite the current literature?

Yes

If applicable, is the statistical analysis and its interpretation appropriate?

Partly

Are all the source data underlying the results available to ensure full reproducibility?

Partly

Is the study design appropriate and is the work technically sound?

No

Are the conclusions drawn adequately supported by the results?

Yes

Are sufficient details of methods and analysis provided to allow replication by others?

No

Reviewer Expertise:

chronic liver disease, portal hypertension

I confirm that I have read this submission and believe that I have an appropriate level of expertise to state that I do not consider it to be of an acceptable scientific standard, for reasons outlined above.

# Response

**Published:** 2023-08-27

**Authors:** Lamine Hamzaoui

**Affiliations:** Mohamed Taher Maamouri Hospital, Tunisia

1. The major concern is the lack of definition of cutoff values of StE to define CHE as the authors didn’t use any reference test. So, we don’t know whether the cutoff values (based on healthy patients) used to classify patients as having CHE or not were right. Thus, neither of the study's objectives were properly met.

**Response:** Thank you for this pertinent comment. Indeed, the lack of a standard reference test may be a limitation of our study, but as we clearly state in the discussion: "the use of PHES and StE, alone or in combination, has been shown to be equivalent for screening for HEC, indicating that a single test with StE is sufficient for screening for HEC.33"

 With regard to threshold values, as with all tests, it is vital to establish local thresholds, based on healthy subjects from the general population, which was done in our study and in all other studies using this tool (recent reference example:

 Cunha-Silva M, Neto FLP, de Araújo PS, Pazinato LV, Greca RD, Secundo TML, Imbrizi MR, Monici LT, Sevá-Pereira T, Mazo DF. EncephalApp Stroop Test validation for the screening of minimal hepatic encephalopathy in Brazil. Ann Hepatol. 2022 Jan-Feb;27(1):100543. doi: 10.1016/j.aohep.2021.100543. Epub 2021 Sep 24. PMID: 34571266.)

 2. In addition, the sample size was small and almost 61% of patients recruited were not included, mainly due to illiteracy.

**Response:**

 2.1. The sample size (n=142) was well above the statistically required number of patients.

 The minimum population size was determined by the Schwarz formula: n= (i² x p x q) / α²

 i = 95% confidence level (i = 0.95)

 p = prevalence of hepatic encephalopathy in cirrhotics is estimated at 0.3 based on a previous study (ref A)

 q = 1-p (q= 0.7)

 α = margin of error at 5% (α = 0.05)

 n = (0.9025 x 0.7 x 0.3)/0.0025= 75,81

 REF A: Fallahzadeh MA, Rahimi RS. Hepatic Encephalopathy: Current and Emerging Treatment Modalities. Clin Gastroenterol Hepatol. 2022 Aug;20(8S):S9-S19. doi: 10.1016/j.cgh.2022.04.034. PMID: 35940731.

 2.2. There are several reasons for the high rate of illiteracy in our study. Firstly, the illiteracy rate is high in Tunisia (17,7% in 2022). According to figures from the National Institute of Statistics, the illiteracy rate among women and men in communal areas has reached 12.9%, while it has risen to 29.5% in non-communal areas. Moreover, the illiteracy rate rises as the average age increases, reaching 2.8% for the 10-14 age group, while it reaches 79.8% among the elderly (aged 80 and over). REF B 

 However, our population tends to be older, from rural and disadvantaged areas, like most of the patients treated in Tunisian public hospitals, which explains the high illiteracy rate and therefore the non-inclusion rate.

 REF B: https://www.courrierinternational.com/article/societe-en-tunisie-les-2-millions-danalphabetes-representent-une-bombe-retardement-sociale

 3. In the section “Prevalence of CHE”, it’s said CHE is more frequent in women… but there are no statistical tests proving it (no p-value).

**Response:** Prevalence of CHE wasn’t affected by gender (p=0.413). The sentence « CHE is more frequent in women » was deleted

 4. In table 3, it’s not very clear the difference between local prevalence of CHE and global prevalence of CHE, please clarify this.

**Response:** The table reports the prevalence of CHE in different regions. It shows that  the rates in the hospitals of Tunis (the capital) were higher than the other regions.

# Reviewer report: Reviewer response for version 1

**DOI:** 10.5256/f1000research.133680.r141643

**Published:** 2022-08-16

**Authors:** Rym Ennaifer

**Affiliations:** 1 Department of Gastroenterology, Mongi Slim La Marsa Hospital, Tunis, Tunisia

This is a well designed multicentric cross sectional study. The methods are well detailed, and the number of patients included is sufficient. Covert encephalopathy is a topical subject in hepatology and difficult to diagnose. The diagnostic method (stroop test) studied is interesting but needs validation.

 Concerning the end of the discussion “Our study is the first one estimating the prevalence of CHE in Tunisia”, actually it is not the first Tunisian study, and here is the reference to cite: Encéphalopathie hépatique minime: un diagnostic précoce pour améliorer le pronostic 

 Minimal hepatic encephalopathy: A better diagnostic to improve prognostic ^1^

 In this study, we used 5 diagnostic tests: Psychometric hepatic encephalopathy score » (PHES): digit symbol test, number connection test A, number connection test B, serial dotting test et line tracing test and the prevalence was 44%. It is very important to cite Tunisian numbers+++ and it was one of the earliest studies about covert encephalopathy in the literature.

Is the work clearly and accurately presented and does it cite the current literature?

Yes

If applicable, is the statistical analysis and its interpretation appropriate?

Yes

Are all the source data underlying the results available to ensure full reproducibility?

No source data required

Is the study design appropriate and is the work technically sound?

Yes

Are the conclusions drawn adequately supported by the results?

Yes

Are sufficient details of methods and analysis provided to allow replication by others?

Yes

Reviewer Expertise:

Hepatology

I confirm that I have read this submission and believe that I have an appropriate level of expertise to confirm that it is of an acceptable scientific standard.

## References

[1] : [Minimal hepatic encephalopathy: a better diagnostic to improve prognostic]. *Presse Med* .2014;43(5) : 10.1016/j.lpm.2013.09.016 e127-33 10.1016/j.lpm.2013.09.016 24703736

# Response

**Published:** 2023-08-27

**Authors:** Lamine Hamzaoui

**Affiliations:** Mohamed Taher Maamouri Hospital, Tunisia

- Prevalence of CHE in the other tunisian study was precised and the study was cited as a reference
